# Multiscale Hierarchical Surface Patterns by Coupling Optical Patterning and Thermal Shrinkage

**DOI:** 10.1021/acsami.0c22436

**Published:** 2021-03-23

**Authors:** Hamidreza Daghigh Shirazi, Yujiao Dong, Jukka Niskanen, Chiara Fedele, Arri Priimagi, Ville P. Jokinen, Jaana Vapaavuori

**Affiliations:** † Department of Chemistry and Materials Science, Aalto University School of Chemical Engineering, Kemistintie 1, 02150 Espoo, Finland; ‡ Département de Chimie, Université de Montréal, C.P. 6128, Succursale Centre-Ville, Montréal, Quebec, Canada H3C 3J7; § Smart Photonic Materials, Faculty of Engineering and Natural Sciences, Tampere University, Korkeakoulunkatu 10, FI-33720 Tampere, Finland

**Keywords:** hierarchical surfaces, wrinkling instability, surface relief gratings, azopolymers, tunable wetting

## Abstract

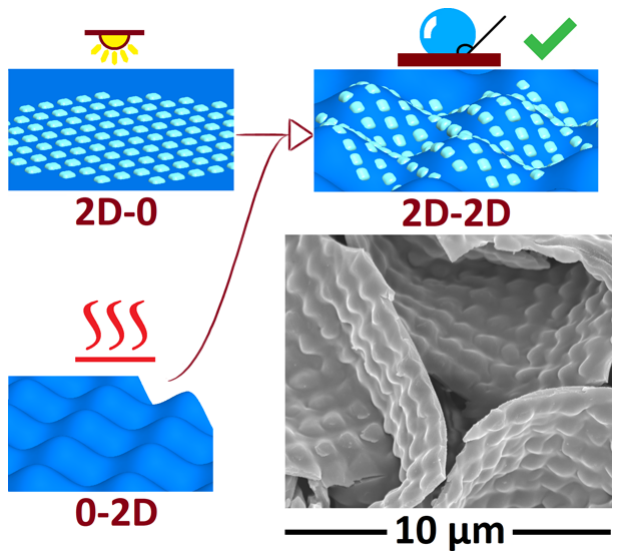

Herein, a simple hierarchical surface patterning method is presented by effectively combining buckling instability and azopolymer-based surface relief grating inscription. In this technique, submicron patterns are achieved using azopolymers, whereas the microscale patterns are fabricated by subsequent thermal shrinkage. The wetting characterization of various topographically patterned surfaces confirms that the method permits tuning of contact angles and choosing between isotropic and anisotropic wetting. Altogether, this method allows efficient fabrication of hierarchical surfaces over several length scales in relatively large areas, overcoming some limitations of fabricating multiscale roughness in lithography and also methods of creating merely random patterns, such as black silicon processing or wet etching of metals. The demonstrated fine-tuning of the surface patterns may be useful in optimizing surface-related material properties, such as wetting and adhesion, producing substrates that are of potential interest in mechanobiology and tissue engineering.

## 1 Introduction

Precisely tailoring the topography of a surface yields intriguing surface properties, such as superhydrophobicity,^1^ self-cleaning,^2,3^ drag reduction in fluid flow,^4−6^ high adhesion surfaces,^7^ antireflection coatings,^8,9^ and structural colors.^10^ These effects are often found in nature, e.g., in shark skin,^4^ lotus leaf,^3^ or butterfly wings,^11^ and emerge from the structural hierarchy built into these biological materials.^12,13^ Surfaces with topographical modulations are of great interest in cell biology for their ability to influence cell morphology and behavior.^14^ Within the tissue, cells interact with multiscale hierarchically assembled topographies, from protein conformation all the way up to the intricate functional assembly of fibrillar structures. Such features can be reproduced in vitro in the form of, e.g., pillars and pits, grooves, and ridges, to allow topographic control on cell fate.^15,16^ Driven by the fundamental quest for the artificial fabrication of these useful functionalities and related commercial interest, finding efficient large-area methods for structurally hierarchical materials is of uttermost importance.^13^

A simple way to produce surface undulations in the length scales spanning from micrometers to millimeters is to expose a double layer of materials with sufficiently mismatched mechanical properties to tensile or compressive forces.^17,18^ When the critical buckling threshold is exceeded, a sinusoidal buckling pattern emerges, the dimensions of which depend on the elastic modulus of the materials, the thickness of the thinner layer, and the magnitude of the disturbing force.^19^ This type of fabrication method has already been used, for instance, to create highly stretchable gold electrodes,^20^ protein preconcentration nanochannels,^21^ cell adhesion platforms,^22,23^ and anisotropically wetting surfaces.^24^

At a length scale of an order of magnitude smaller than the previous examples, azobenzene-containing polymers are known to undergo micrometer-scale mass transport under a light polarization/intensity interference pattern, providing a facile all-optical method for large-area surface patterning.^25−29^ Due to the nondestructive nature of this method, even reversible surface structuring has become feasible.^30−32^ The resulting structured surfaces have found applications in, e.g., photonics^26,28,33^ and wettability,^34,35^ as well as in cell adhesion and orientation.^36−38^ To further expand the hierarchical surface wrinkles, various attempts have been reported on coupling the all-optical azopolymer pattern inscription technique with other methods, such as soft lithography,^39,40^ thermally induced fluidization,^39^ and external mechanical stimuli.^30^

Both buckling and azopolymer-based surface patterning methods differ significantly from the commonly used, top-down strategies of fabricating surface microstructures through conventional micro- and nanofabrication, such as ultraviolet (UV) or electron-beam (e-beam) lithography.^41^ Neither of these methods is particularly well suited for fabricating large areas (>cm^2^ sized) of submicrometer features since UV lithography lacks the resolution and e-beam lithography is extremely slow and expensive for large-area patterning. On the other hand, some microfabrication methods, for example, maskless plasma etching and wet etching, have been used to create submicron roughness in silicon,^9^ polymers,^42,43^ and metals.^44^ These roughness fabrication methods are suitable for the large-area fabrication of two-dimensional (2D) isotropic roughness but typically not for fabricating surfaces with anisotropic properties. Moreover, the combination of these conventional microfabrication methods is rarely straightforward.

Herein, we combine the buckling-based material fabrication and all-optical surface patterning of azopolymers for the preparation of hierarchical grating-on-a-grating structures. The buckling instability of the materials and their layer thickness allows easy production of undulating structures at 10 μm length scales, while the periodicity of the optically inscribed azopolymer surface relief gratings allows patterning at submicron length scales. By combining these two techniques, we establish a method for hierarchically structured surfaces for which the two length scales of the structure can be tuned independently. In contrast to previous studies, including those with the coupling of the optical patterning method to other patterning methods in the literature,^29,30,39,40,45−48^ the combination presented in this work provides dimensionally tunable hierarchical wrinkles with a diversified range of patterns using a widely accessible, simple, rapid, versatile, and inexpensive thermal shrinkage method. The aforementioned characteristics of this approach, along with not requiring conventional lithographic and microfabrication tools and cleanroom conditions, reduce the barriers and enable widespread use in general academic laboratories. Also, taking into account the conformal nature of both patterning methods, relatively large hierarchically structured areas can be prepared in a one-step process. Furthermore, both isotropic two-dimensionally structured surfaces and anisotropic directional hierarchical surfaces can be fabricated, providing a route toward functional surfaces with demanding optical, adhesion, and wetting properties. These functional surfaces are potentially intriguing for applications of structural colorization and outcoupling enhancement in organic light-emitting diode (OLED) devices.^40,49,50^ Wetting characterization of these surfaces shows that the chosen surface structure predictably leads to either isotropic or anisotropic wetting properties.

## 2 Results and Discussion

### 2.1 Diversity of the Produced Hierarchical Structures

**Figure 1:**
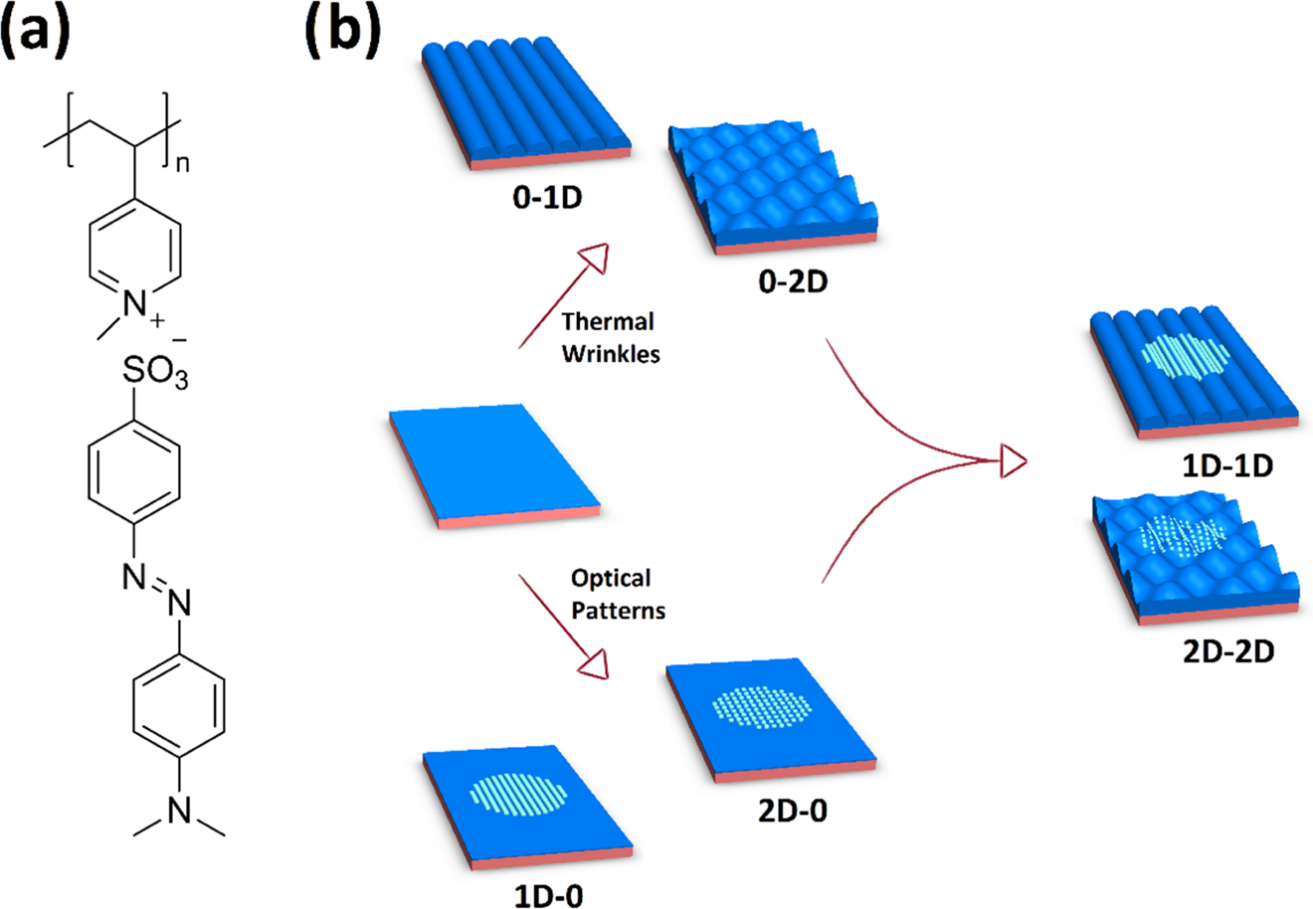
(a) Chemical structure of the supramolecular polymer–azobenzene complex and (b) schematic of the fabrication process.

To be able to combine the two different patterning methods—the all-optical surface patterning and the wrinkling instability upon stretching/contraction of a double layer—two materials with mismatching properties, prestretched poly(styrene) (PS) and a supramolecular polymer–azobenzene complex consisting of quaternized poly(4-vinylpyridine) and methyl orange (the chemical structure is shown in Figure 1a), were chosen. More importantly, this choice was motivated by the need of finding a polymer–azobenzene complex, which is known for its high all-optical surface patterning efficiency, and also of a glass transition temperature (*T*_g_) higher than that of PS.^51,52^ Since it is well known that the optically inscribed azopolymer surface patterns can be erased either optically^31,32,53^ or by heating the azopolymers above the glass transition temperature,^54,55^ the significantly higher *T*_g_ of the polymer–azobenzene complex ensures that the wrinkling instability and optical surface patterning can be considered as orthogonal methods.

**Figure 2:**
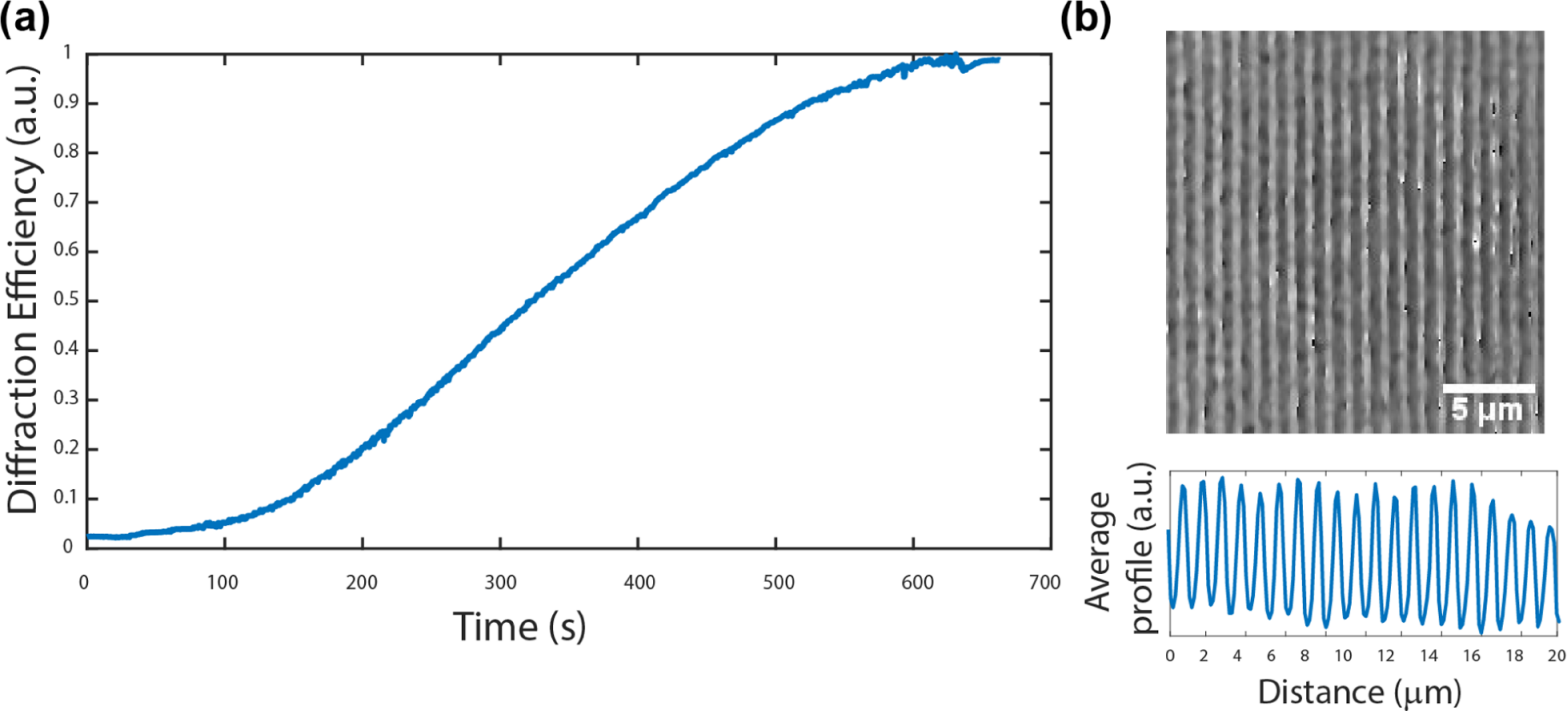
(a) Diffraction efficiency curve for the inscription of an azopolymer (SRG) with ca. 1 μm period. (b) Digital holographic microscopy (DHM) phase image of the same SRG and the average surface profile.

Figure 1b summarizes our fabrication method leading to hierarchically structured surfaces. First, double layers of PS and the azopolymer were prepared by spin coating, and optical patterns were inscribed to the azopolymer layers using a sinusoidal interference pattern of a circularly polarized 488 nm laser beam and a Lloyd’s mirror interferometer. As expected, based on the literature and monitored by the diffraction efficiency curve growth in Figure 2a, surface relief grating (SRG) formation with a modulation depth of >100 nm was observed within a 10 min inscription period. This figure also highlights the additional advantage of the chosen azomaterial allowing faster inscription of high-modulation depth gratings as compared to many other supramolecular polymer–azobenzene complexes.^25^

The inscribed gratings are known to be temporally stable for several years at ambient conditions, yet they can be erased thermally^54,55^ or optically,^31,32,56^ thus bringing about post-modification possibilities. The sinusoidal profile of the photoinscribed SRG was measured by digital holographic microscopy (DHM), a quantitative phase imaging technique.^57,58^ DHM measures both the amplitude and the phase of the light reflected from the sample. It reconstructs the surface modulation from the hologram created by a reference beam and a beam reflected by the sample surface. In Figure 2b, the sinusoidal modulation depth (>100 nm) is extracted from hologram’s phase reconstruction data and it is well consistent with the atomic force microscopy (AFM)-measured surface relief grating depths for the same polymer–azobenzene complex.^51^ It is well known that the inscription process could have been suspended at an earlier stage, should precise control over modulation depth bring some advantages for the final application. Subject to the fundamental diffraction limit of light, the azopolymer surface patterning offers lots of possibilities in controlling the grating periodicity and the modulation depth—by intensity, polarization, and inscription time. Furthermore, more complex surface structures can be written by overlaying multiple different interference patterns,^54^ employing different methods to produce interference and near-field patterns,^28,57,59−62^ and combining steps of selective surface inscription and erasure.^31^

**Figure 3:**
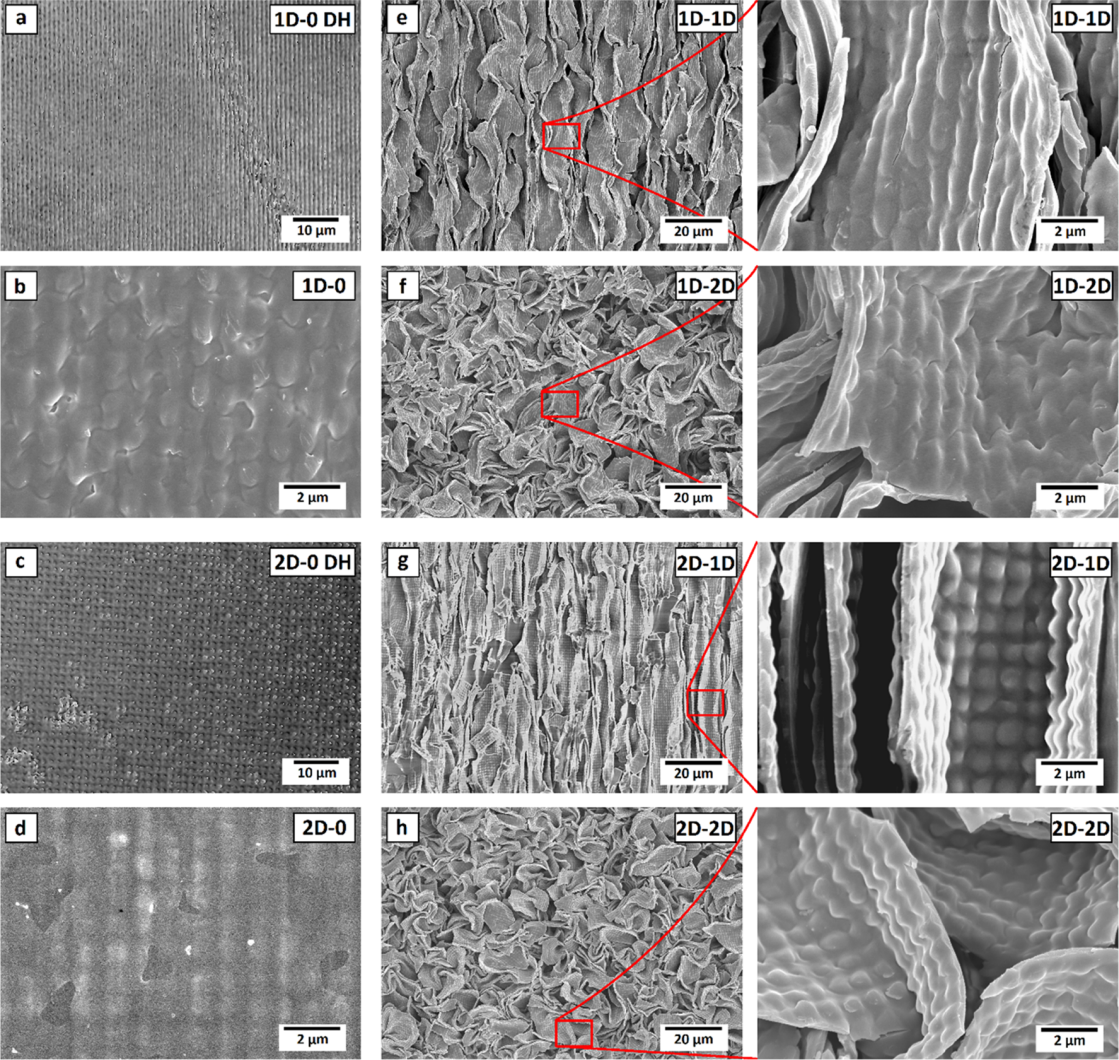
Digital holography (DH) and SEM images of samples with (a, b) 1D and (c, d) 2D optical patterns and after inducing (e–g) 1D and (f–h) 2D thermal wrinkles. (Small-scale grating pattern periodicity otherwise 1 μm, for (c), 1.5 μm).

Next, the double layers containing azopolymer surface patterns were subjected to a short (4 min) heat treatment at 150 °C to form hierarchical structures. The wrinkling structure resulting from the buckling instability in samples with no directional restriction results in 2D hierarchical surface patterns, whereas one-dimensional (1D) surface wrinkles become feasible by restricting the shrinkage to one dimension by clamping. As shown in Figure 3, this shrinking process conserves the original optical grating pattern (illustrated in Figure 3a–d). The anisotropic 1D larger-scale wrinkles containing 1D and 2D smaller-scale optical patterns are shown in Figure 3e,g, respectively. For 2D shrunken patterns, the shrinkage of the samples has successfully led to the generation of isotropic eggshell patterns, containing the previously inscribed anisotropic (Figure 3f) and isotropic (Figure 3h) optical patterns. An average size of ca. 10–15 μm for the repeating periodical structure can be estimated based on the scanning electron microscopy (SEM) images.

The buckling instability causing these large-scale surface structures is known to depend on both the mechanical properties of the two materials and the thickness of the azopolymer layer.^19,63^ Thus, the controlled tunability of these structures would be easy to achieve by producing films of different thicknesses, varying the spin-coating parameters, such as the polymer concentration and the rotation rate. The combination of two different length scales and two different dimensionalities (1D or 2D) for both length scales leads to a large number of possible surface topographies. This variability would be difficult to achieve using methods that are based on random roughness, such as maskless wet or dry etching. The overall three-dimensionality of the structures makes them difficult to replicate by lithographic methods even if two consecutive lithography steps were to be utilized for the two length scales. Finally, the rapid process (both the optical inscription and heat treatment being completed in the timescale of minutes) and facile fabrication in cm^2^ and larger size makes this method competitive against, e.g., two-photon three-dimensional (3D) printing, which could reach the same size scales and three dimensionalities.

To classify the samples, we adopt a naming convention of XD–YD, also marked in Figure 3, in which X refers to the dimensionality of the azopolymer optical grating and Y refers to the dimensionality of the buckling-instability-induced patterns. Unless otherwise stated, the periodicity of the optical gratings is fixed to 1 μm. In case the periodicity differs from 1 μm, it is specified in the name.

### 2.2 Wetting Properties

The effect of the various surface textures on the wetting properties of the samples was evaluated by contact angle goniometry, the results of which are shown in Table 1. We chose to characterize the wetting properties after the addition of a hydrophobic fluoropolymer coating. The reason for using the coating was twofold. First, the contact angles of the planar sample without the coating were an advancing contact angle of 80 ± 2° and a very low receding contact angle of 14 ± 3°. Therefore, for both 2D–1D and 2D–2D samples, the receding contact angles to all directions were close to 0, too low to be measured by goniometry. The advancing contact angles on the uncoated surfaces exhibited some slip-stick characteristics, making the contact angle measurement unreliable. Second, using the coating standardizes the surface chemistry right before the wetting characterization to eliminate all inadvertent changes to surface chemistry that might occur during the processing of various types of samples. This allows us to interpret the results in terms of the structures only, which is the focus of this manuscript.

**Table 1:**
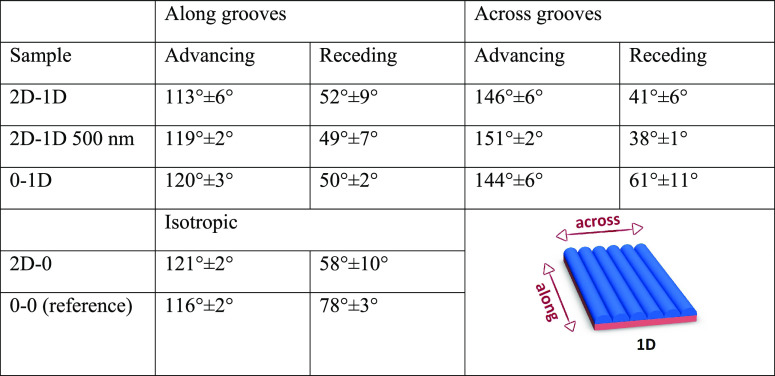
Contact Angles of the Samples after Hydrophobic Coating^a^

a Note: the values given are the average ± standard deviation of three measurements.

The hydrophobic fluoropolymer coating was deposited by plasma-enhanced chemical vapor deposition on all samples before the wetting characterization. The nominal thickness of the coating on a flat surface is 40 nm, and the thickness is less on more vertical walls. Because of this, the coating does not significantly alter the topography; the SEM images in Figure 3 are of the samples with the coating. The 0–0 sample is a planar polystyrene sample with the hydrophobic coating, with the advancing contact angle well in excess of 90° (the receding contact angle is <90°). The optical pattern alone (2D–0) leads to a small but clearly detectable effect on the wetting properties. The increased roughness leads to an increase in the advancing contact angle from 116 ± 2° to 121 ± 2° and a decrease in the receding contact angle from 78 ± 3°to 58 ± 10°. The contact angle hysteresis thus increases from 38 to 67°. Surface roughness is widely understood to be one of the main causes of contact angle hysteresis.^64^

**Figure 4:**
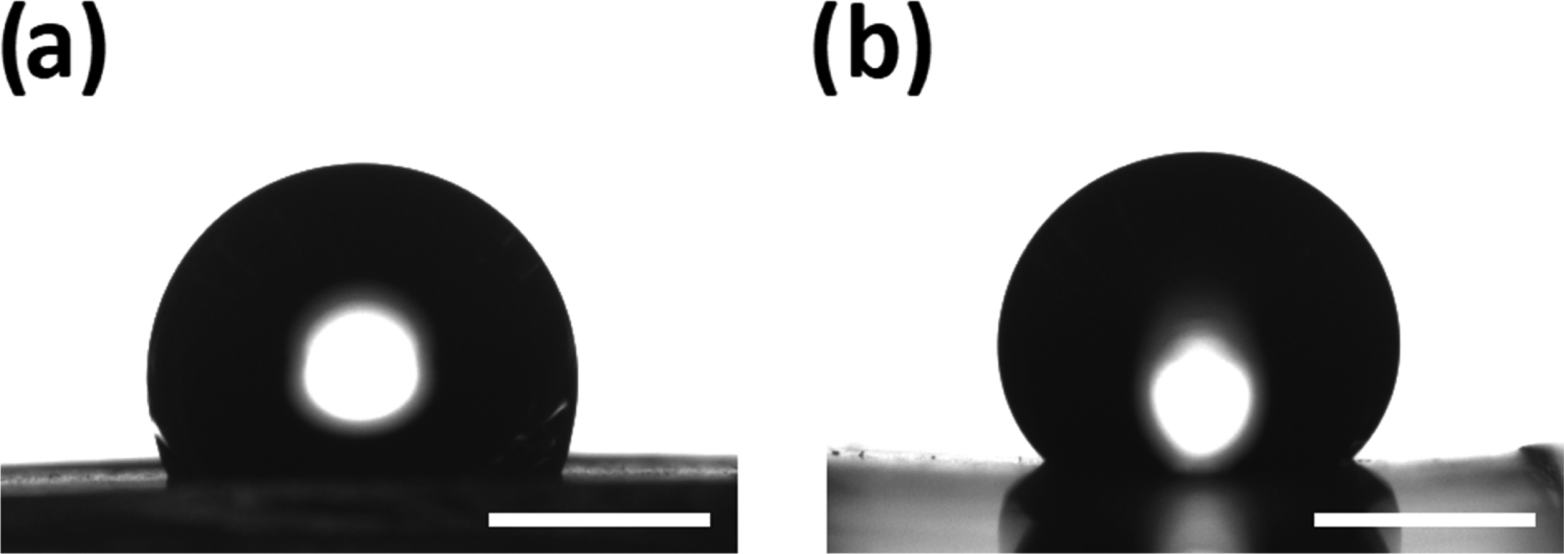
Optical images of droplets on the 2D–1D sample when the contact line is advancing (a) along the grooves and (b) across the grooves. The scale bar (bottom right) is 1 mm.

The samples with 1D thermal shrinkage patterns exhibited anisotropic wetting where the droplets more easily spread to the direction along the grooves compared to spreading across the grooves. This leads to asymmetrical droplets where the contact angle depends on the viewing angle. Figure 4 shows the same droplet on the 2D–1D sample taken from two viewing directions, one along the grooves (Figure 4a) and one across the direction perpendicular to the grooves (Figure 4b). The contact angle of the contact line spreading along the grooves is shown in Figure 4a, and the corresponding case of the contact line spreading across the grooves is shown in Figure 4b. The goniometry showed that the advancing contact angles of the 2D–1D, 2D–1D 500 nm, and 0–1D samples were 115–120° along the grooves and 145–150° across the grooves, showing an anisotropy of approximately 30° in the advancing contact angle. For the receding contact angle, the anisotropy was not as clear. The receding contact angles for the 2D–1D and 2D–1D 500 nm samples were similar, around 50° along the grooves and 40° across the grooves for an anisotropy of 10°. However, the 0–1D sample had receding angles of 50° along the grooves and 61° across the grooves for an anisotropy of 11° but to the opposite direction. The variability within the samples was also higher in the receding angles, so we conclude that with these surfaces, the structures add more anisotropy to the advancing contact angle compared to the receding contact angle. The relatively high contact angle hysteresis values of all thermally structured samples show that in all of the cases, the droplet was in the Wenzel^65^ wetting state, where the liquid contacts all of the surface instead of an air pocket being trapped between the surface and the droplet as is the case with the Cassie^66^ wetting state.

Anisotropic wetting on grooves is widely known in the micrometer^67,68^ and submicron^69^ scales. Chen et al. showed that on 25 μm wide microfabricated grooves made out of poly(dimethylsiloxane) (PDMS), the contact angle was approximately 145° when spreading across the grooves and approximately 125° when spreading along the grooves.^67^ Contact angle goniometry had insufficient sensitivity to distinguish between the 0–1D, 2D–1D 1 μm, and 2D–1D 500 nm surfaces as all showed the same anisotropic wetting behavior. The effect of the optical pattern likely leads to fine-tuning of the contact angle also for the groove structures, similar to what was shown with the 0–1D samples, but goniometry is limited in its sensitivity for small differences.

The 2D–2D samples had high advancing contact angles of ≈155° and extremely low receding contact angles. The 2D–2D samples could not be reliably measured with goniometry due to their smaller size (<1 cm) resulting from thermal shrinkage in both directions and the overall curvature of the samples. It was clear, however, that these surfaces had a combination of high contact angles and high hysteresis, which is called the petal effect.^70^ Overall, wetting analysis shows that the fabricated hierarchical surfaces can be used to tune the wetting properties over a range of different isotropic and anisotropic behaviors.

### 2.3 Demonstration of Gradient Hierarchical Surfaces

**Figure 5:**
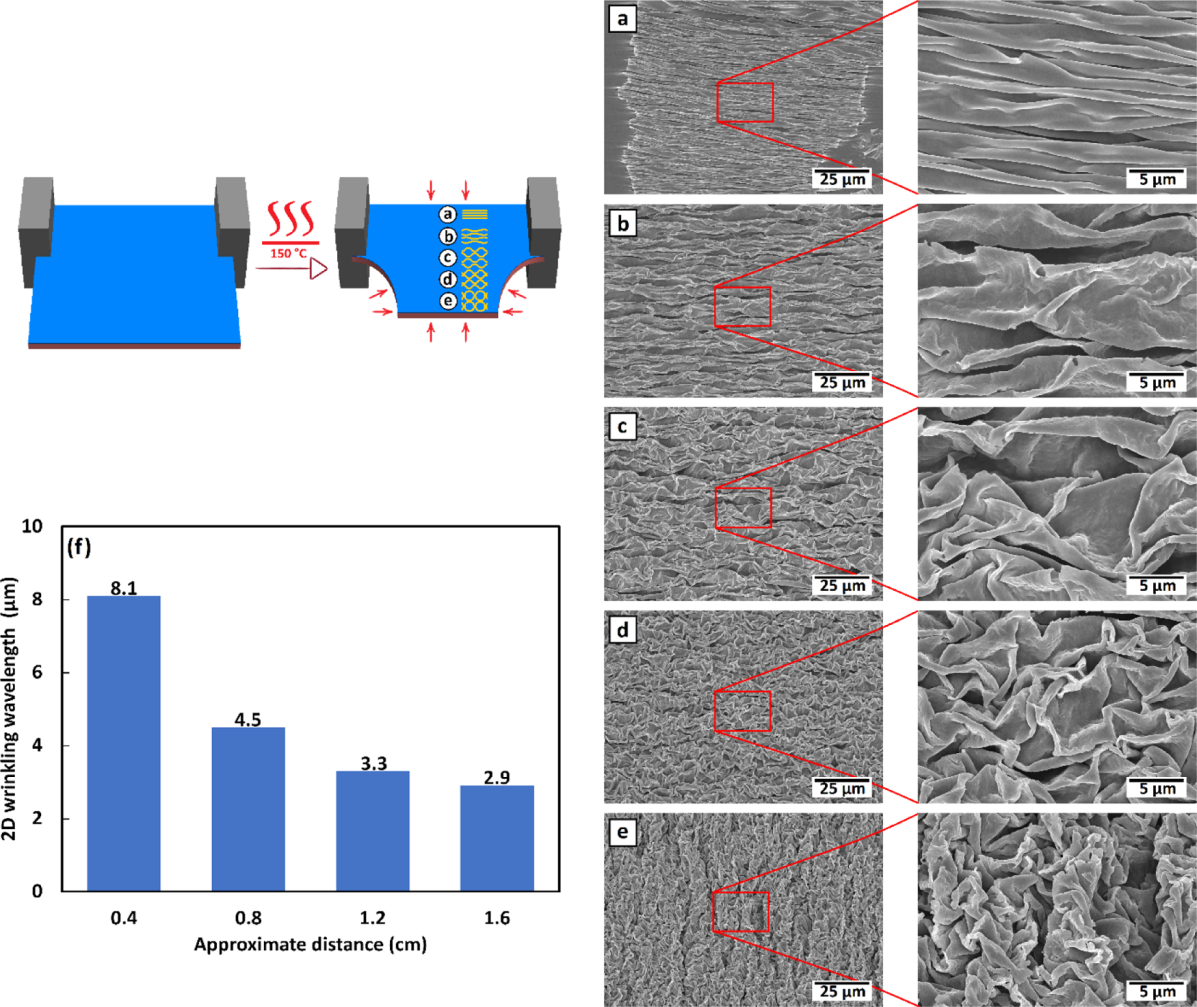
Schematic representation of the selective spinning technique, as well as (a–e) SEM images of the resulting gradient surface with ca. 0.4 μm distances, demonstrating a gradual transition between 1D and 2D larger-scale wrinkles, and (f) the wavelength periodicity of 2D wrinkling as a function of deviation from the 1D region, derived from FFT analyses.

The dimensionality of the larger-scale structures can also be gradually varied by selectively clamping the substrates before the heat treatment. Pinning the two adjacent corners and leaving the other two corners free, as illustrated in Figure 5, will result in a gradual transition between the 1D- and 2D-structured surface patterns over a distance of approximately 1 cm. These types of hierarchical gradient surfaces may turn out to be useful, for instance, in tissue engineering applications for testing the preferential surface attachment of proteins,^71^ mechanotaxis,^22^ stem cell differentiation,^72,73^ facilitating inkjet printing,^74^ and for producing ratcheting motion of droplets.^75^ While gradients can be created using many methods by adding extra complications to the fabrication process, here we achieve the gradients with no extra steps by just changing the attachment points of the sample during the thermal shrinkage.

Figure 5f provides a quantitative demonstration of the wrinkling wavelength characteristics of the unstrained regions, presenting the gradual intervention of isotropic wrinkling. For this means, 2D fast Fourier transform (FFT) analysis on the SEM images with lower magnification (Figure 5a–e) has been performed to determine the evolution from 1D to 2D wrinkling of the surface structures. The presented histogram shows the obtained wrinkling (peak-to-peak) wavelengths, deviating from the strained 1D region, where the characteristic wrinkling wavelength of the unstrained region gradually decreases till the ultimate 2D structure is produced.

## 3 Conclusions

We demonstrated a simple fabrication method for hierarchical structures for which both directionality and dimensions can be independently tuned at two different length scales. The micrometer-scale control is induced upon shrinking of a double-layered structure consisting of a prestretched polymer substrate coated with a photoresponsive azopolymer film. The control of dimensions at the scale of the light wavelength is based on the capability of the azopolymer to undergo a light-induced motion under the illumination of either the intensity or the polarization interference pattern of light that can activate the photoisomerization of azobenzene. The timescale of the total process is in the order of tens of minutes. The large library of hierarchically different surface topographies that can be achieved with a simple process is the big advantage of the fabrication scheme proposed as compared to conventional microfabrication processes. The wrinkled systems proposed herein provide an interesting combination of anisotropic 1D and isotropic 2D topographies that point toward possible applications in, e.g., wetting and adhesion control, but also lie in a biologically relevant range. Such multiscale structures could be used, for instance, in the study of stem cell differentiation, tissue formation, or even developmental biology.

## 4 Experimental Procedure

### 4.1 Synthesis of the Azopolymer

#### 4.1.1 Synthesis of Poly(1-methyl-4-vinylpyridin-1-ium iodide)

The quaternization was done as reported by Zhang et al.^51^ P4VP (*M*_n_ = 4000 g/mol, 500 mg, 4.8 mmol repeating units) was dissolved in nitromethane (7 mL) at 45 °C and iodomethane (1.7 g, 12.0 mmol) was added to the solution. After 5 days, the polymer was precipitated in diethyl ether and washed with diethyl ether. Poly(1-methyl-4-vinylpyridin-1-ium iodide) (P4VP-4k-Q, 1.02 g) was obtained after drying in vacuo. The degree of functionalization (>99%) was determined by ^1^H nuclear magnetic resonance (NMR) from the integrals of the hydrogens in the aromatic ring and the methyl group on the quaternized nitrogen. ^1^H NMR (400 MHz, D_2_O, TMS, δ ppm): 2.23 (−C*H*_2_−), 2.95 (−C*H*−), 4.27 (C*H*_3_–N−), 7.73 (−C*H*−), and 8.57 (−C*H*−).

P4VP (*M*_n_ = 50 000 g/mol, 508 mg, 4.8 mmol repeating units) was quaternized similarly using nitromethane (7 mL) and iodomethane (1.7 g, 12.0 mmol) at 45 °C, yielding in 1.88 g of poly(1-methyl-4-vinylpyridin-1-ium iodide) (P4VP-50k-Q). ^1^H NMR (400 MHz, D_2_O, TMS, δ ppm): 2.17 (−C*H*_2_−), 2.83 (−C*H*−), 4.27 (C*H*_3_–N−), 7.69 (−C*H*−), and 8.57 (−C*H*−).

#### 4.1.2 Complexation of Poly(1-methyl-4-vinylpyridin-1-ium iodide) with Methyl Orange

The complexation of methyl orange with poly(1-methyl-4-vinylpyridin-1-ium iodide) was conducted as described by Zhang et al.^51^ P4VP-4k-Q (0.503 g, 2.16 mmol repeating units) was dissolved in water (8 mL). Methyl orange (805 mg, 2.46 mmol) was dissolved in warm dimethyl sulfoxide (DMSO) and added to the polymer solution at 45 °C. The polymer precipitated during the addition of the methyl orange solution, and DMSO was added until the polymer dissolved. The solution was kept at 45 °C for 1 h, after which it was dialyzed against deionized water for 8 days while changing the water daily. The polymer (0.80 g) was finally recovered by freeze drying. ^1^H NMR (400 MHz, D_2_O, TMS, δ ppm): 1.87 (−C*H*_2_−), 3.04 (−C*H*_3_), 4.19 (−C*H*_3_), 6.88 (−C*H*−), 7.05 (−C*H*−), 7.65 (−C*H*−), 7.76 (−C*H*−), and 8.66 (−C*H*−).

The complexation of P4VP-50k-Q (0.502 g, 2.16 mmol repeating units) was conducted in a similar manner, yielding in 0.59 g of the product. ^1^H NMR (400 MHz, D_2_O, TMS, δ ppm): 1.69 (−C*H*_2_−), 3.01 (−C*H*_3_), 4.10 (−C*H*_3_), 6.75 (−C*H*−), 7.74 (−C*H*−), and 8.58 (−C*H*−).

### 4.2 Preparation of the Double-Layered Films

The prestretched PS substrates were cut as 2.5 cm × 2.5 cm, and thereafter plasma cleaned for 1 min at 50% power by a Henniker Plasma HPT-100. The solutions of P4VP-methyl orange types B and C were prepared by dissolving in DMSO at a 10 wt % concentration while heating. Thereafter, the solutions of P4VP-methyl orange B and C were spin coated on the prestretched PS substrates, performed by a Laurell WS-650-23B. The spin-coating protocol was accelerated for 5 s to reach 2000 rpm and then kept for 30 s.

### 4.3 Preparation of the Optical Surface Patterns

The samples were patterned with interference lithography in Lloyd’s mirror configuration. An optically pumped semiconductor laser with a continuous wave (CW) output of 488 nm with a 2 W maximum output power (Genesis CX 488-2000, Coherent Inc., Santa Clara, CA) was spatially filtered, circularly polarized, and projected at the interface between the sample and a mirror with an intensity of 300 mW/cm^2^. The interference pattern of light on the films induced the formation of sinusoidal surface relief gratings (SRG), replicating the polarization light pattern. The pattern period Λ is given by eq 1
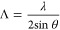
1where λ is the laser wavelength and ϑ (set to 14° for 1 μm and 29° for 500 nm) is the angle between the incident beam and the mirror. 2D patterns were obtained by rotating the sample 90° after the first inscription. The irradiation time was chosen from the samples with 1 μm period by monitoring the evolution up to the saturation level of diffraction efficiency, defined as the ratio between the power of the first-order diffracted beam and the initial beam, with a low-power He–Ne laser (633 nm). In Figure 2a, an exemplar curve of normalized diffraction efficiency for 1 μm grating is shown.

### 4.4 Preparation of the Hierarchical Surface Patterns

The optically patterned samples were cut in 2 cm × 2 cm square pieces and then freely shrunk in an oven for 4 min at 150 °C to attain 2D hierarchical surface patterns. 1D surface patterns were prepared by restricting the shrinkage along one of the directions. Accordingly, the samples were cut into 1 cm × 2 cm stripes, where the end sides of the stripes were clamped to retain their position. The samples were then kept at 150 °C for 8 min.

### 4.5 Preparation of the Gradient Surface Patterns

The gradient hierarchical surfaces were prepared by combining the abovementioned processes. One side of the sample, with a size of 2 cm × 2 cm, was mounted on the clamps, whereas the other side was facing no constraint toward shrinkage. The shrinkage has been similarly performed at 150 °C for 8 min.

### 4.6 Surface Characterization

#### 4.6.1 Contact Angle Measurements

The samples were made hydrophobic by applying a plasma-enhanced chemical vapor deposition (Oxford Plasmalab 80+) fluoropolymer coating. The parameters of the deposition were 250 mTorr pressure, 50 W power, 100 sccm CHF_3_ flow, and 6 min deposition time.

The contact angles were measured by sessile droplet goniometry using the needle-in-droplet method. The advancing contact angles were measured by increasing the volume of the droplet from 1 to 3 μL at a rate of 0.1 μL/s. The receding contact angles were measured by decreasing the volume of the droplet from 3 to 0 μL at a rate of 0.1 μL/s.

#### 4.6.2 Digital Holographic Microscopy

Surface relief gratings were characterized via reflection digital holographic microscopy (DHM R-2100, Lyncee tec., Lausanne, CH). The DHM records the hologram of the surface and reconstructs its phase and intensity with a reconstruction algorithm. In this way, the surface profile can be semiquantitatively evaluated (Figure 2b).

#### 4.6.3 Scanning Electron Microscopy (SEM)

A 10 nm layer of 80/20 Au–Pd was first sputtered on the samples. Then, the surfaces were analyzed using SEM (Hitachi S-4700).

#### 4.6.4 2D Fast Fourier Transform (FFT) Analysis

The analysis was conducted in the image processing software of ImageJ by converting the estimated structure factors to distance, representing the wavelengths in real space, for SEM images with lower magnification shown in Figure 5a–e.
